# Comparison of the Users’ Attitudes Toward Cannabidiol on Social Media Platforms: Topic Modeling Study

**DOI:** 10.2196/34132

**Published:** 2023-01-11

**Authors:** Yongjie Li, Xiangyu Yan, Zekun Wang, Mingchang Ma, Bo Zhang, Zhongwei Jia

**Affiliations:** 1 School of Public Health, Peking University Beijing China; 2 School of Cyberspace Security, Beijing University of Posts and Telecommunications Beijing China; 3 Center for Intelligent Public Health, Institute for Artificial Intelligence, Peking University Beijing China; 4 Center for Drug Abuse Control and Prevention, National Institute of Health Data Science Beijing China

**Keywords:** cannabidiol, drug policy, latent Dirichlet allocation, social media, sentiment analysis

## Abstract

**Background:**

As one of the major constituents of the cannabis sativa plant, cannabidiol (CBD) is approved for use in medical treatment and cosmetics because of its potential health benefits. With the rapid growth of the CBD market, customers purchase these products, and relevant discussions are becoming more active on social media.

**Objective:**

In this study, we aimed to understand the users’ attitudes toward CBD products in various countries by conducting text mining on social media in countries with different substance management policies.

**Methods:**

We collected posts from Reddit and Xiaohongshu, conducted topic mining using the latent Dirichlet allocation model, and analyzed the characteristics of topics on different social media. Subsequently, a co-occurrence network of high-frequency keywords was constructed to explore potential relationships among topics. Moreover, we conducted sentiment analysis on the posts’ comments and compared users’ attitudes toward CBD products on Reddit and Xiaohongshu using chi-square test.

**Results:**

CBD-related posts on social media have been rapidly increasing, especially on Xiaohongshu since 2019. A total of 1790 posts from Reddit and 1951 posts from Xiaohongshu were included in the final analysis. The posts on the 2 social media platforms, Reddit and Xiaohongshu, were categorized into 7 and 8 topics, respectively, by the latent Dirichlet allocation model, and these topics on the 2 social media were grouped into 5 themes. Our study showed that the themes on Reddit were mainly related to the therapeutic effects of CBD, whereas the themes on Xiaohongshu concentrated on cosmetics, such as facial masks. Theme 2 (CBD market information) and theme 3 (attitudes toward CBD) on Reddit had more connections with other themes in the co-occurrence network, and theme 3 and theme 1 (CBD therapeutic effects) had a high co-occurrence frequency (22,803/73,865, 30.87%). Meanwhile, theme 1 (CBD cosmetics) on Xiaohongshu had various connections with others (169,961/384,575, 44.19%), and the co-occurrence frequency of theme 4 (CBD ingredients) and theme 1 was relatively prominent (27,128/49,312, 55.01%). Overall, users’ comments tended to be positive for CBD-related information on both Reddit and Xiaohongshu, but the percentage was higher on Xiaohongshu (82.25% vs 86.18%; *P*<.001), especially in cosmetics and medical health care products.

**Conclusions:**

The CBD market has grown rapidly, and the topics related to CBD on social media have become active. There are apparent differences in users’ attitudes toward CBD in countries with different substance management policies. Targeted CBD management measures should be formulated to suit the prevalence of CBD use of each country.

## Introduction

### Background

Cannabidiol (CBD) is a nonintoxicating principal constituent of the cannabis sativa plant discovered in 1940 [1]. In recent years, CBD has been widely used in cosmetics, food supplements, beverages, electronic cigarette liquids, and prescription pharmaceuticals. There are dozens of different forms of CBD products on the market, such as oils, capsules, edibles, and so on. Unlike tetrahydrocannabinol (THC), which is the main psychoactive component of cannabis, relevant studies have shown that CBD is a relatively safe and nonaddictive substance [2]. Moreover, there is evidence that CBD has variety of pharmacological effects on the receptor system, producing analgesic, anti-inflammatory, antianxiety, and antipsychotic effects [3]. Relevant clinical studies have also shown that CBD has the potential to treat anxiety disorders, panic disorders, and pain [4-7]. In addition, Tóth et al [8] suggested that CBD may be helpful in treating some skin symptoms, such as dryness and itching. Nevertheless, owing to the lack of large-scale randomized controlled trials, the clinical effectiveness and safety of CBD are uncertain [9,10] and its potential side effects have not yet been confirmed [11].

In the context of the global legalization of cannabis, government policies regarding CBD are inconsistent. In the United States, most states allow CBD for medical use and residents can purchase it from pharmacies or on the web [12]. However, products containing CBD with therapeutic claims are tightly regulated by the Food and Drug Administration, and except Epidiolex, no products have been approved by the Food and Drug Administration [13]. As the second largest CBD market, most European countries have legalized CBD products (THC not exceeding 0.2%), and those products with medical claims must be authorized as medical products [14,15]. Most customers in Europe are still free to purchase CBD products on the web. The acceleration of global cannabis legalization will lead to a further boom in the CBD market in the United States and European countries. In contrast, in countries such as China and Singapore, illicit drugs are strictly regulated by the government, including heroin, cannabis, and its derivatives. The legality of CBD has not been approved, and its use in food, medicine, and cosmetics is prohibited, as well as web-based or offline trade [16,17]. However, because of the fast-growing interest in lifestyle upgrades, customers in these countries have also begun to pay attention to CBD products [18].

With the explosive growth in the popularity of CBD products, related topics on social media are steadily increasing. People, especially adults aged between 18 and 35 years, are keen to share their feelings on social media. Moreover, users can comment on topics that they are interested in to express their thoughts and interact with others. Social media is an essential channel for CBD product marketing. Users often read related promotions when surfing on social media. Approximately 38% of customers discovered CBD through internet searches [19]. Keeping abreast of topics on social media will assist us in understanding the trends in CBD products, which have a substantial impact on people’s behavior [20]. Previous studies have demonstrated the potential of social media in perceiving product trends in CBD [21,22]. However, these studies focus on social media data in the United States and European countries, where CBD has been approved for use and sale.

### Objectives

Propelled by the spread of information and promotion by celebrities, CBD products are landing on social media in China. As China implements strict drug regulatory policies, the popularity of CBD products on social media in China will bring new challenges to current policies, and there is no relevant research to date. This study aimed to extract and analyze topics on social media in China and other countries to explore users’ attitudes toward CBD products and compare the differences with social media users. We hope to provide suggestions to policy makers to cope with the rapid development of the CBD market.

## Methods

### Data Collection and Preprocessing

**Figure 1:**
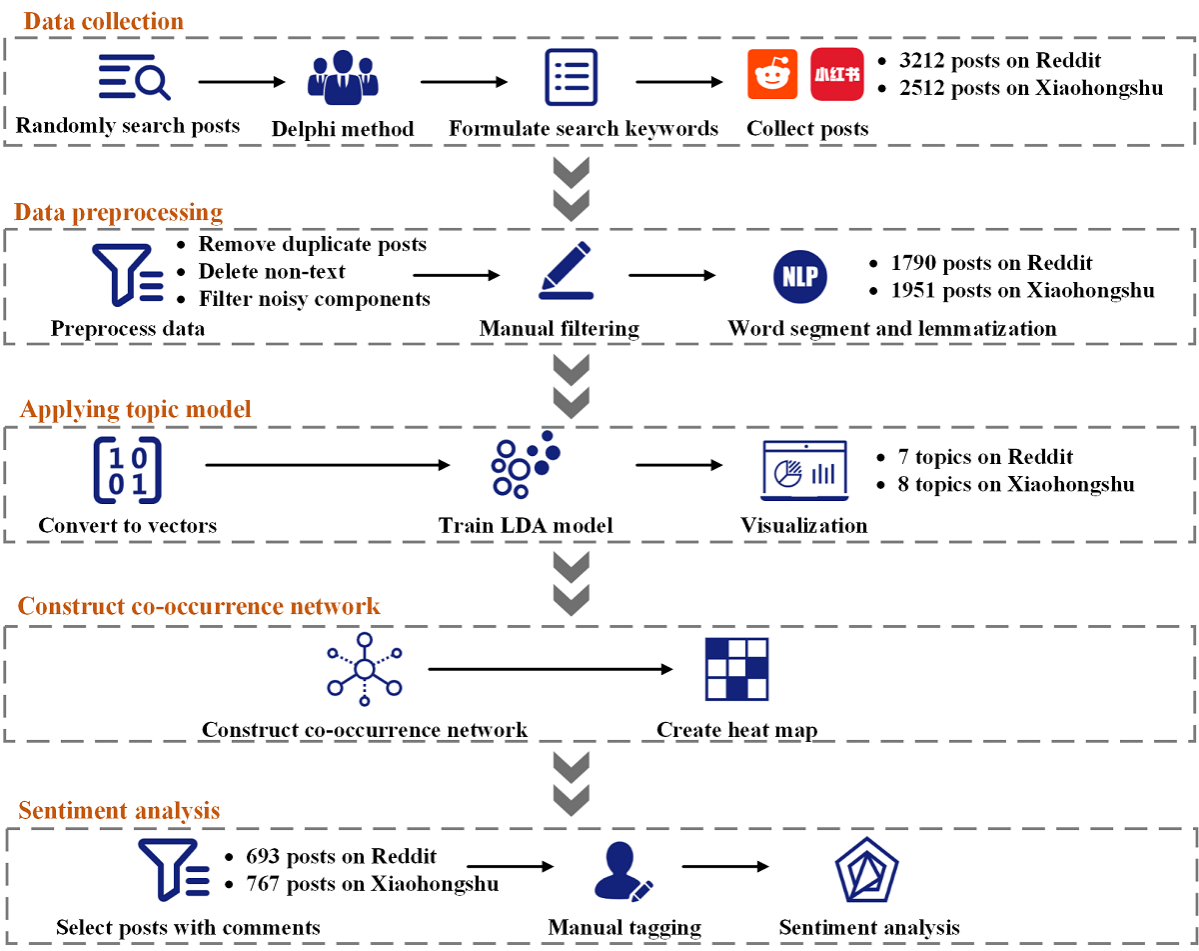
Flowchart of the study. LDA: Latent Dirichlet Allocation.

First, we chose 2 popular social media platforms, Reddit and Xiaohongshu. Reddit is an English language–centric social community that includes a variety of topics, and its user base is primarily Western countries [23]. A report on the demographics of Reddit users showed that their locations were mainly from the United States, Australia, Brazil, Canada, Denmark, Norway, and other countries, which were also representative countries where CBD products were used. High school and college education accounted for the highest proportions, and the users’ age ranged from 18 to 49 years [24,25]. As one of China’s fastest growing social media apps, Xiaohongshu has approximately 100 million active users, most of whom are from China [26]. Xiaohongshu encourages its users to post different contents of their lives, and users can interact based on interests, such as fashion, food, and many other aspects. A study emphasized that posts on Xiaohongshu had a substantial influence on the purchasing behavior of Chinese consumers [27]. Considering that marijuana is strictly regulated in China, related posts are not allowed to be published. As Xiaohongshu is a platform connecting domestic and overseas users, CBD-related posts highlighted the current attitude of domestic users. Therefore, these posts on Xiaohongshu, as a representative, were used to analyze attitudes of users in China. We formulated a keyword list to retrieve CBD-related posts from the 2 social media platforms (Figure 1). In the beginning, we randomly searched 100 posts containing *CBD* from the 2 social media platforms and then 2 experts independently reviewed these posts and selected the appropriate keywords. Finally, the Delphi method was used to determine the completed search keyword list (Textbox 1) [28]. We searched all posts and the related comments from social media based on the keyword list we formulated until May 31, 2021. Each post included the serial number that was used to filter duplicates, titles, detailed content, and published dates. The comments on the post were composed of text content and comment date (Multimedia Appendix 1, Figure S1). As unstructured data contained considerable noise, all the original data were further processed to facilitate subsequent analysis. Duplicate posts resulting from different search keywords were removed with their comments based on the serial number. Then, nontext data (eg, video and picture) or blank content in the posts or comments were deleted. Subsequently, unnecessary sentence components (eg, emoji, hyperlink, and punctuations) and meaningless words (common stop words in English and Chinese) in the text were removed. Moreover, regular expressions were used to eliminate nonalphanumeric characters. In addition, we noticed that there were still a few irrelevant posts in the data set. Two researchers independently filtered unrelated posts. If there was a discrepancy, a third researcher determined the final outcome. Finally, for the Chinese posts on Xiaohongshu, we executed a word segment on the sentence using Jieba 0.42. In addition, we performed lemmatization on Reddit posts to extract more textual information using NLTK 3.5. After the above preprocessing, all the remaining data constituted the corpora of Reddit and Xiaohongshu.

**Search keywords on Reddit and Xiaohongshu.**

**Search keywords on Reddit**

- cbd oil
- cbd cream
- hemp oil
- cbd hemp oil
- cbd oil anxiety
- cbd for pain
- cbd vape
- cbd benefits
- cbd liquid
- cannabidiol
- cbd cannabidiol
- cannabidiol oil
- cannabidiol cbd oil
- aceite cannabidiol
- cannabidiol capsules
- cannabidiol gummies
- cannabidiol products

**Search keywords on Xiaohongshu**

- cbd 油
- cbd 护肤
- cbd 面霜
- cbd 成分
- cbd 产品
- cbd 食品
- cbd 软糖
- cbd 化妆品
- cbd 胶囊
- cbd 焦虑
- cbd 失眠
- cbd 水乳
- cbd 疼痛
- cbd 抑郁
- cbd 大麻油
- cbd 烟
- cbd 电子烟

### Data Analysis

#### Applying Topic Model

The topic model is a statistical algorithm for discovering the latent semantic structures of large corpora that can offer insights into ways we can better understand the main idea of the text. Latent Dirichlet allocation (LDA) is an unsupervised generative probabilistic method for modeling extensive text, resulting in different topics and document clusters [29]. Before applying LDA to the topic model, the text in the corpora was converted to a vector of term counts using Scikit-learn, a Python library (Figure 1). Perplexity was used as the criterion for evaluating the effectiveness of the LDA model; the lower the perplexity, the better the model predicts [30]. To determine the appropriate number of topics in the corpus of documents, the 5-fold cross-validation was applied in LDA model training. We randomly shuffled the corpus and divided it into 5 groups, 4 of which were training data sets, and the remaining one was a validation data set [31,32]. The candidate number of topics ranged from 2 to 15. We also calculated coherence measures to evaluate the candidate number of topics. Topic coherence measures scored a single topic by measuring the degree of semantic similarity between high-scoring words in the topic. Coherence value (CV) was selected as the metric in this study, and the higher value indicated optimal topic coherence [33,34]. Then, during each training iteration, we calculated and compared the perplexity and coherence scores of different candidate topic numbers in the validation data sets and presented these results in a scatter plot. Therefore, the final number of topics in the corpus was determined using a combination of perplexity and coherence. After the LDA model training was completed, we visualized the generated topics and related keywords. In the visualization layout, topics were represented in the form of bubbles, and each bubble represented one topic. The distance between the bubbles was an approximation of the semantic relationship between the topics. The overlap of bubbles indicated that there were similar parts between the topics. On the basis of the outcomes in the layout and combined with a better expression of the semantics of these topics, we merged similar topics (overlapping and closer in the distance) extracted by the LDA model into new themes.

#### Construct Co-occurrence Network of High-Frequency Keywords

A co-occurrence network was used to provide an intuitive expression of potential relationships between topics within corpora, and it was the collective interconnection of various high-frequency keywords based on their paired presence within the posts in the corpora. The top 10 high-frequency keywords in each theme were regarded as representative high-frequency keywords. We then formed pairs of these high-frequency keywords (Figure 1). In this study, we defined “co-occurrence” as a pair of keywords appearing in the same post. For example, “cannabidiol” (keyword in theme 1) and “experience” (keyword in theme 3) appeared in the post, so this pair (cannabidiol-experience) was said to “co-occur.” Moreover, we counted the frequency of co-occurrence of high-frequency keywords in the corresponding corpora. We then constructed a co-occurrence network diagram of high-frequency keywords, where the nodes with colors represented the keywords of themes and the thickness of the edges indicated the frequency of co-occurrence in the corresponding corpora. In addition, the corresponding heatmaps were created to provide the relationships between various themes intuitively. In the co-occurrence heatmap, the number represented the frequency of co-occurrence between different themes, and the pie chart indicated the proportion of 1 theme in all themes in each row. This work was performed using Gephi 0.9.2 (Gephi Consortium) and Seaborn modules in Python 3.6 (Python Software Foundation).

#### Sentiment Analysis

Social media is one of the most important ways for people to obtain information, and sentiment analysis can help understand a person’s opinion about a particular subject or topic based on the comments on posts [35]. Therefore, we selected posts with comments to form subcorpora and performed sentiment analysis on them (Figure 1). As the comments were feedback on the content of posts, we first paid attention to the content discussed in these posts, and we found that most of them were divided into 3 subjects: cosmetics, food, and medical health care products. We assigned 2 researchers with medical backgrounds to annotate the subject of posts with labels. The 2 researchers were asked to independently classify the posts and reach a consensus on the final label results. If there was an inconsistency, an expert would participate and ensure the final agreement. Next, we arranged for 2 other researchers to independently annotate the comments based on the detailed text to determine sentiment polarity (positive or negative). Positive comments referred to those messages that users liked or accepted in the content of posts, and negative comments were just the opposite. The process of determining positive or negative comments was consistent with the above process. According to the sentiment polarity annotation results on the comments, we set a sentiment classification label for each post. For example, the posts that had more positive comments than negative ones would be classified as positive posts and vice versa. Finally, we drew a scatter plot of sentiment polarity. The horizontal coordinates indicated the subject classification of the post, and the vertical coordinates indicated the number of comments (positive posts used the number of positive comments and negative posts used the number of negative comments). The differences in sentiment distribution between the 2 social media platforms were verified using the chi-square test executed in SPSS (version 22.0; IBM Corp).

### Ethics Approval

This study was approved by the Peking University Institutional Review Board (IRB00001052-16016).

## Results

### Basic Statistics Information

**Figure 2:**
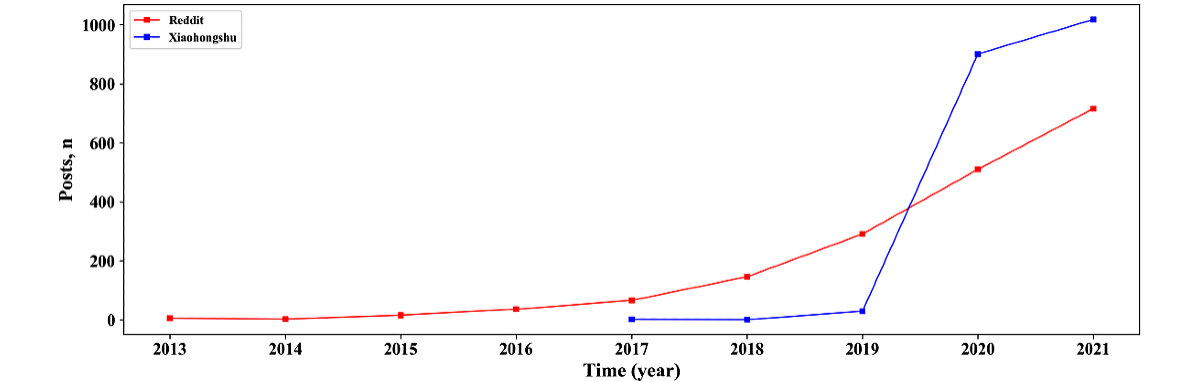
The number of posts on Reddit and Xiaohongshu per year.

By the end of May 31, 2021, 3212 Reddit posts and 2512 Xiaohongshu posts were collected. After preprocessing, 1790 and 1951 posts were included in the statistical analyses. According to the statistics of the number of posts by years, it was apparent that there was a rising trend of CBD-related posts, both on Reddit and Xiaohongshu (Figure 2). In particular, compared with posts on Reddit, the attention paid to CBD on Xiaohongshu was hysteretic, and there had been explosive growth since 2019.

### Topic Modeling Result

After 5-fold cross-validation, perplexity was the lowest when the topic number was 7 on the Reddit corpus (199.34-201.91). Likewise, the highest CV using CV metric ranged from 0.63 to 0.67 with a topic number of 7. Therefore, 7 topics were identified from the posts on Reddit (Multimedia Appendix 1, Figures S2 and S3). The 7 topics were grouped into 5 themes: CBD product treatment and effect (theme 1, including 2 topics), market information related to CBD products (theme 2, including 2 topics), user attitudes toward CBD products (theme 3, including 1 topic), products and forms related to CBD (theme 4, including 1 topic), and consumer-oriented CBD product format (theme 5, including 1 topic). The keywords for each theme with the percentage of the given documents are summarized in Table 1. It showed that theme 1 was the most popular, accounting for 47.64% (43,197/90,676) of the keywords. Moreover, in theme 3 (17,565/90,676, 19.37%), many users expressed their desire to try CBD products, as advertisements on the CBD were readily available on similar websites. In addition, it was clear that there were various forms of CBD products in the consumer market, which were distinctive from the themes of Xiaohongshu. Perplexity and coherence varied for different values of the number of topics. On the basis of the perplexity and coherence of the Xiaohongshu corpus, we determined 8 as the optimal number of the topics with the minimum perplexity (137.15-140.70) and maximum coherence (0.64-0.68; Multimedia Appendix 1, Figures S4 and S5). We grouped these 8 topics into 5 themes (Table 2). The most obvious characteristic was that Xiaohongshu’s themes were concentrated on cosmetics field. Theme 1 (including 3 topics) had the highest percentage (25,750/55,097, 46.74%) and was CBD-related cosmetics categories and functions, such as facial masks, creams, and essences. In addition, the themes on Reddit and Xiaohongshu both involved brands of CBD. However, there were not only domestic brands on Xiaohongshu but also international brands from the United States and Europe (theme 2, including 2 topics; 10,001/55,097, 18.15%). Moreover, theme 3 (including 1 topic) and theme 4 (including 1 topic) introduced the effect evaluation of CBD products and the raw materials of CBD cosmetics, respectively. Theme 5 (including 1 topic) described medical health care products with therapeutic benefits claims, and a similar theme also appeared in Reddit.

**Table 1:** Topic classification and keywords on Reddit corpora.

Classification and topics		Keywords	Values, %
**Theme 1: CBD^a^ production treatment and effect**			
	Topic 1: CBD production treatment	pain, anxiety, body, treatment, patients, use, reduce, sleep, disorders, cancer	25.56
	Topic 2: Multiformat CBD products effect	hemp, make, gummies, benefit, plant, oil, cannabidiol, extract, high, health	22.08
**Theme 2: Market information related to CBD products**			
	Topic 3: Company information of CBD	company, products, base, business, market, release, sell, service, live, develop	11.43
	Topic 4: Brand and report of CBD	hemp, brand, share, report, sales, industry, grow, research, offer, hold, price	8.61
**Theme 3: Attitudes toward for CBD products**			
	Topic 5: Attitude to use CBD products	like, know, want, feel, work, try, think, start, experience, really, need, good	19.37
**Theme 4: Products and forms related to CBD**			
	Topic 6: Raw materials and other products	cannabis, marijuana, medical, extract, weed, capsule, flower, smoke, food	6.82
**Theme 5: Consumer-oriented CBD product format**			
	Topic 7: Product formats and distribution channels	vape, bottle, liquid, spectrum, cream, free, online, shop, pure, website, order	6.13

^a^CBD: cannabidiol.

**Table 2:** Topic classification and keywords on Xiaohongshu corpora.

Classification and topics		Keywords	Values, %
**Theme 1: CBD^a^-related cosmetics categories and functions**			
	Topic 1: Skin care cosmetics	skin (皮肤), sensitive (敏感), ingredient (成分), cream (面霜), skin care products (护肤品), lotion (水乳), absorb (吸收), oil skin (油皮), protect (护理)	19.33
	Topic 2: Benefits of CBD mask	facial mask (面膜), repair (修护), stay up late (熬夜), hydrating (补水), emollient (舒缓), serum (精华液), fight against acne (消炎), antioxidant (抗氧化), emergency (急救)	15.67
	Topic 3: Benefits of other CBD cosmetics	acne prone (痘痘), oil-control (控油), moisturize (保湿), purify (淡化), resurfacing (清爽), nutritious (舒缓), whitening (美白), antiwrinkle (抗皱), active (活化)	11.74
**Theme 2: CBD-related cosmetics brands and research**			
	Topic 4: Study on the effectiveness of CBD	function (作用), efficacy (功效), research (研究), cell (细胞), health (健康), control (抑制), discovery (发现), stimulate (刺激), balance (平衡)	9.32
	Topic 5: CBD-related cosmetics brands	products (产品), brand (品牌), industry (工业), extract (提取物), western (欧美), formulation (配方), America (美国), price (价格), domestic (国产)	8.83
**Theme 3: Effect evaluation of CBD products**			
	Topic 6: Cosmetics effect evaluation	feel (感觉), really (真的), particular (特别), smell (味道), comfy (舒服), recommend (推荐), good (不错), try (试试), share (分享)	12.11
**Theme 4: Main ingredients of CBD cosmetics**			
	Topic 7: Ingredient introduction	Cannabis (大麻), ingredient (成分), essence (精华), vitamin (维生素), essential oil (精油), extract (提取), add (添加), plant (植物), natural (天然)	11.98
**Theme 5: Declared disease treatment effect**			
	Topic 8: CBD treatment of diseases	sleep (睡眠), anxiety (焦虑), relieve (缓解), insomnia (失眠), stress (压力), emotion (情绪), pain (疼痛), adjustment (调节), improve (改善)	11.02

^a^CBD: cannabidiol.

### Co-occurrence Network of High-Frequency Keywords

**Figure 3:**
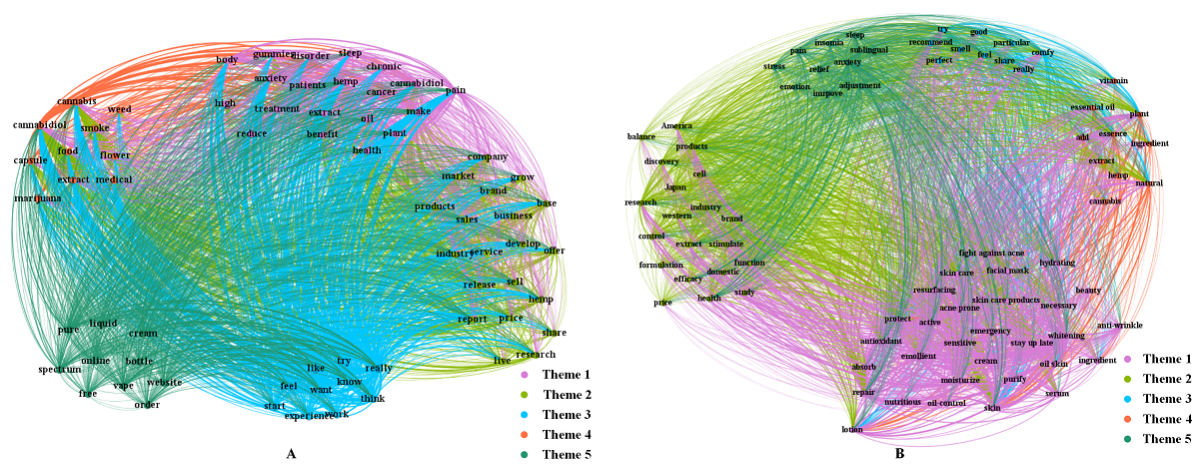
Co-occurrence network of high-frequency keywords on Reddit and Xiaohongshu. (A) The nodes represent the keywords of the themes on Reddit. The edge between two nodes (source node and target node) indicates that two keywords appear in the same post, and the thickness of the edges indicates the frequency of co-occurrence in the posts. The color of the edge is consistent with that of the source node. Theme 1: CBD production treatment and effects. Theme 2: market information related to CBD products. Theme 3: attitudes toward CBD products. Theme 4: products and forms related to CBD. Theme 5: consumer-oriented CBD product format. (B) The nodes represent keywords of the themes on Xiaohongshu. The edge between two nodes (source node and target node) indicates that two keywords appear in the same post, and the thickness of the edges indicates the frequency of co-occurrence in the posts. The color of the edge is consistent with that of the source node. Theme 1: CBD-related cosmetic categories and functions. Theme 2: CBD-related cosmetic brands and research. Theme 3: effect evaluation of CBD products. Theme 4: main ingredients of CBD cosmetics. Theme 5: declared disease treatment effect. CBD: cannabidiol.

**Figure 4:**
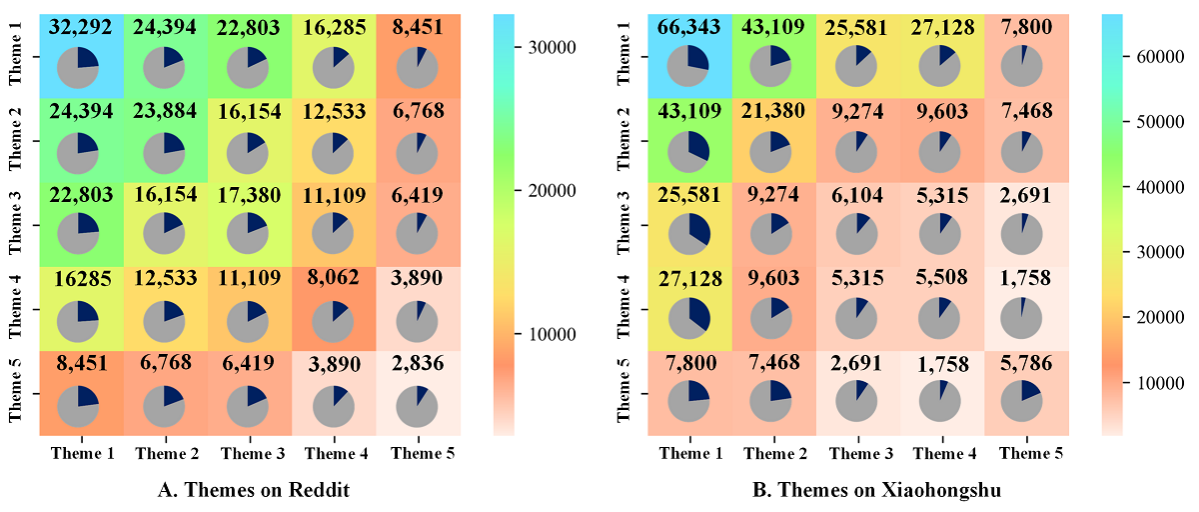
Co-occurrence heatmap of high-frequency keywords on Reddit and Xiaohongshu. The number is the frequency of co-occurrence between different themes, and the pie chart (blue part) indicates the proportion of one theme in all themes in each row. (A) The themes on Reddit—theme 1: CBD production treatment and effect, theme 2: market information related to CBD products, theme 3: attitudes toward CBD products, theme 4: products and forms related to CBD, and theme 5: consumer-oriented CBD product format. (B) The themes on Xiaohongshu—theme 1: CBD-related cosmetics categories and functions, theme 2: CBD-related cosmetic brands and research, theme 3: effect evaluation of CBD products, theme 4: main ingredients of CBD cosmetics, and theme 5: declared disease treatment effect. CBD: cannabidiol.

For the themes on Reddit, there were wide connections between the different themes. Obviously, theme 2 had the most connections with other themes, which was 24.48% (83,733/342,066) followed by theme 3 that accounted for 21.59% (73,865/342,066; Figure 3A). The heatmap of high-frequency keywords showed that theme 3 and theme 1 had a high co-occurrence frequency (22,803/73,865, 30.87%); that is, users were interested in the therapeutic effects of CBD and expressed their thoughts on trying and some even used this type of product for the first time (Figure 4A). This study also showed that there were various forms of CBD products for users to try and choose, which was why the co-occurrence frequency of theme 5 and theme 3 was relatively high (6419/28,364, 22.63%). In contrast, as users in Xiaohongshu shared all kinds of posts about cosmetics, the themes of CBD-related cosmetics (theme 1) categories had various connections with others (169,961/384,575, 44.19%; Figure 3B). Furthermore, unlike the themes of Reddit mentioned above, the themes in Xiaohongshu that claimed the therapeutic effects (theme 5) were simply associated with cosmetics and derivatives of CBD ingredients (theme 4), which was only 6.89% (1758/25,503). Xiaohongshu users were more willing to spend time introducing ingredients in cosmetics, especially those related to CBD. Therefore, the co-occurrence frequency of theme 4 and theme 1 was relatively prominent (27,128/49,312, 55.01%; Figure 4B).

### Distribution of Sentiment Polarity

**Figure 5:**
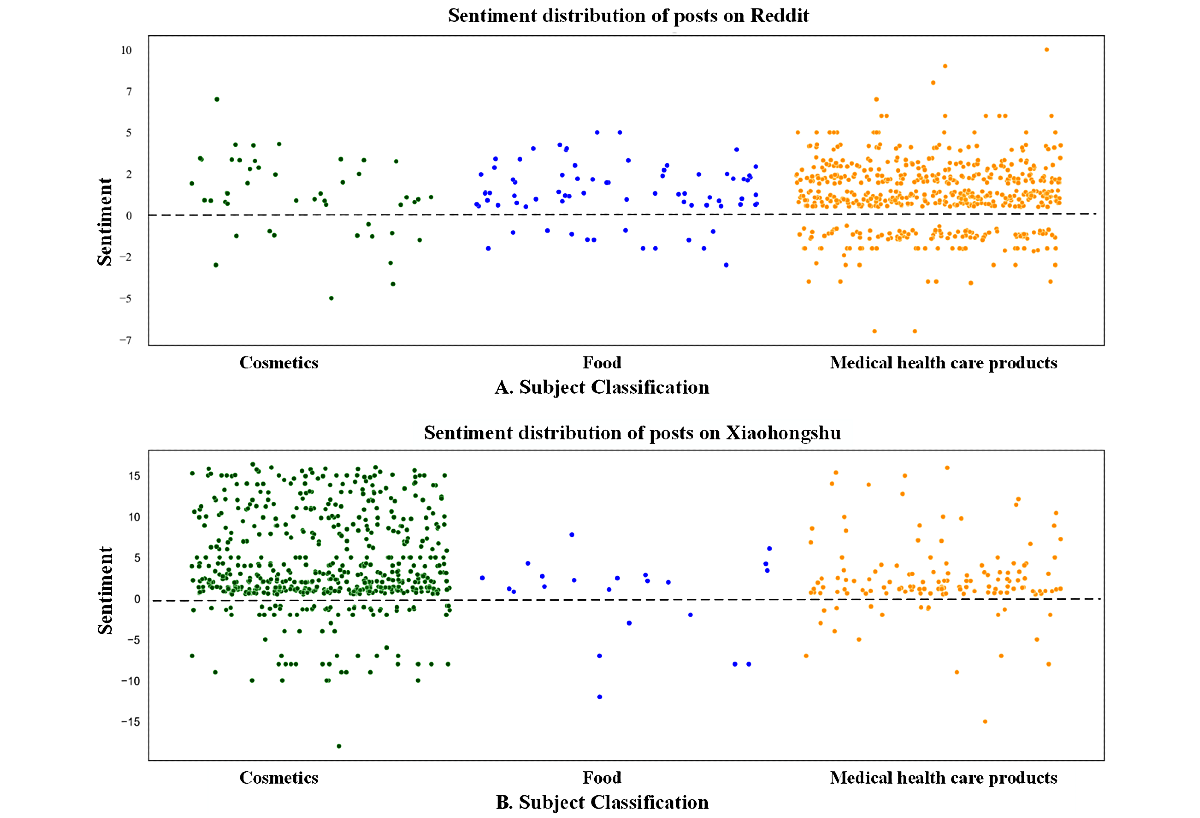
Sentiment distribution of posts on Reddit and Xiaohongshu. The horizontal coordinates indicate the subject classification of the post, and the vertical coordinates indicate the number of comments (positive posts used the number of positive comments, and negative posts used the number of negative comments).

After processing, there were 693 and 767 posts with comments on Reddit and Xiaohongshu, respectively (Figure 1). From the perspective of the classification subject of the posts, it was evident that the posts on Reddit were more focused on medical health care products (571/693, 82.4%), whereas the posts on Xiaohongshu had the highest proportion of posts on cosmetics (605/767, 78.88%; Figure 5). On Xiaohongshu, information about food containing CBD ingredients did not attract much attention (22/767, 2.87%), and discussions about cosmetics in Reddit were also less common (47/693, 6.78%). However, we noted that approximately 2.6% (18/693) of posts on Reddit reported using CBD products as alternative medicines, such as recreational cannabis and painkillers.

Overall, regardless of Reddit or Xiaohongshu, users’ comments tended to be positive for CBD-related information, but the percentage was higher on Xiaohongshu (Reddit vs Xiaohongshu: 82.25% vs 86.18%; *P*<.001). Compared with Reddit, Xiaohongshu also had a higher percentage of positive comments on the subject of cosmetic and medical health care products (74.47% vs 91.57% in cosmetics and 82.84% vs 86.43% in medical health care products; *P*=.02). There was no significant difference in the proportion of posts with positive or negative emotions on the food subject between the 2 social media platforms (Table 3).

**Table 3:** The distribution of sentiment polarity on Reddit and Xiaohongshu.

				Overall						Cosmetics						Food						Medical health care products				
				Value, n (%)		Value, N		*P* value		Value, n (%)		Value, N		*P* value		Value, n (%)		Value, N		*P* value		Value, n (%)		Value, N		*P* value
**Comments**								<.001						.02						.30						.02
	**Reddit**																									
		Positive	540 (82.25)		693				35 (74.47)		47				62 (82.67)		75				443 (82.84)		571			
		Negative	153 (17.75)		693				12 (25.53)		47				13 (17.33)		75				128 (17.16)		571			
	**Xiaohongshu**																									
		Positive	661 (86.18)		767				524 (91.57)		605				16 (72.73)		22				121 (86.43)		140			
		Negative	106 (13.82)		767				81 (8.43)		605				6 (27.27)		22				19 (13.57)		140			

## Discussion

### Principal Findings

This novel study analyzed the posts related to CBD products on social media and compared users’ attitudes toward CBD in countries with different substance management policies. The number of cannabis-related topics on social media was massive, and CBD was one of the emerging topics that attracted more and more attention. Benefiting from the formulated search keywords, the corpus in this study filtered out many data with high-noise levels, which also made the corpus relatively small. Therefore, we could build a more effective topic model to mine users’ attitudes toward CBD products. The results showed that there were differences in the themes that users discussed on social media and their attitudes toward CBD had their own characteristics. Obviously, compared with some Western countries, there was a “vacuum period” when the relevant theme was first available on Xiaohongshu in China. Although China adheres to the strict drug control policy (including cannabis and its derivatives), with the popularity of legalization of medical and recreational cannabis, related themes have become increasingly active on social media in China [36,37]. The text-based topic model analysis in this study provided a reference for cross-cultural research. In traditional cross-cultural studies, qualitative methodologies were prominent and mostly conducted by cultural anthropologists in the form of ethnographies [38]. This required researchers to have a variety of cultural backgrounds, which may otherwise cause research bias. Language independence is commonly presented as one of the advantages of modern machine learning approaches to natural language processing (NLP), and it is an important type of scalability [39]. NLP technology can help us achieve cross-cultural quantitative analysis. In this study, we established an automated topic analysis process. A sequence of NLP functions was used to mine and extract topics from social media texts, and these topics were compared in different cultural contexts.

Social media serves as the vital source of information, as people are willing to share information on social media, and we can mine topic information from user-generated content on social media [40]. Although China is the largest producer of industrial hemp, its strict policy supervision has restricted the application of CBD. Cosmetics with CBD ingredients have attracted consumers, and it has been booming on social media. Even the international brands from the United States and Japan are also sold in the domestic market through e-commerce platforms (eg, Taobao and JD). Not only cosmetics but also some medical health care products that claimed to relieve anxiety or body pain have also appeared on social media in China. As more countries approved CBD for medical purposes, previous studies reported similar trends on other social media in Western countries [22]. Currently, there is a lack of scientific evidence for its effectiveness in treatment, and policy makers should pay attention to this phenomenon. Although the sale of cannabis and its derivatives is banned in China, CBD products for medical use have bypassed the legal supervision under the cover of cosmetics. In addition, many users claimed that taking CBD was good for health and even advocated anticancer effects, which may cause patients to eschew proven treatments and increase the disease burden [15]. Although CBD is touted for its therapeutic properties by consumers, public health professionals should be cautious when recommending CBD products and should ensure that regional and local laws are followed. Health care providers should clearly inform patients of the potential risks, especially in vulnerable groups such as children, older adults, and patients who are chronically ill. Moreover, our study found that users’ attention to the 2 social media platforms was significantly different. Regarding CBD products, Xiaohongshu users posted more frequently about CBD in skin care, whereas Reddit users posted more frequently about the potential medicinal value of CBD products. Therefore, the government needs to strengthen the surveillance of the market and guide consumers to correctly understand CBD to avoid unnecessary risks owing to false propaganda.

There are various novel CBD product forms (eg, vaping and gummies) on Reddit, which are popular among teenagers. We noticed a close connection between this theme (theme 5) and the theme of therapeutic effects (theme 1) on Reddit. When teenagers are exposed to such information on social media, curiosity may drive them to try these CBD products, such as e-cigarettes, to relieve anxiety and further use CBD with high THC levels or even cannabis [41]. Although there were few related themes on Xiaohongshu, China would face the same situation with the rapid growth of the e-cigarette market. Therefore, it is urgent to conduct education and intervention in schools or entertainment venues. In addition, it is necessary to improve the detection of THC in CBD products. A previous study reported several CBD products with excessive THC in the European market [15]. The customs department should strengthen its supervision of the imported e-liquid. On Xiaohongshu, internet celebrities often introduced the main ingredient CBD and its relationship with cannabis when they shared the experience of using cosmetics. However, some of the contents lacked scientific evidence or were fake news. These kinds of fake news on social media mislead people and put public health at risk [42]. Therefore, social media should exercise professional responsibility to ensure that accurate information is published and disseminated. Policy makers need to penalize false or unsubstantiated CBD advertisement claims, especially for CBD foods with unclear efficacy. Any products making claims for therapeutic or medical use must be approved by regulatory authorities upon submission of data. In addition, regulators should strengthen the training of health care providers to ensure the safety and effectiveness of the prescriptions.

Finally, the sentiment polarity of comments on the 2 social media platforms reflected that users’ attitudes toward CBD products differed. The almost overwhelmingly positive comments on cosmetics showed the popularity of CBD cosmetics among Chinese consumers. Most of the comments on Reddit focused on CBD health care products, but the proportion of negative comments was higher than that on Xiaohongshu. In contrast to China’s drug policy, people in the West (ie, the United States and European countries) are free to buy and use medical CBD products. However, the therapeutic efficacy with actual experience may be lower than the advertised therapeutic efficacy, leading to more negative comments on social media. For example, 1 user said that the use of CBD oil did not alleviate body pain at all, and that the illness was finally cured by routine clinical treatment. In addition, the discussion on the legal status of CBD revolves around the question of whether it is a drug or food supplement. Many products were labeled as food to avoid regulation, but current data show that consumers, both in China and the West, are not particularly concerned about it. Overall, users were more positive about CBD products on social media in China, even though CBD is still illegal. As an emerging trend, domestic consumers know little about CBD and do not know enough about its management requirements and use risks, so the government should focus on publicizing CBD-related scientific evidence and management measures. Public health communities need to keep abreast of the latest research and implement evidence-based practice standards and guidelines to reduce the risk of potential misuse of CBD products.

### Limitations

This study has several limitations. Our data were collected from social media, which are mostly used by internet-savvy young people. Therefore, there may be a lack of input from older adults who rarely use social media. However, considering that the primary consumers of CBD products are young people, our study represented most of the target population. In addition, the user demographics of Reddit and Xiaohongshu are not representative of all Western and Chinese populations. It is necessary to investigate more users beyond social media platforms to evaluate actual consumer attitudes. The NLP analysis is correlated with the amount of data, and the relatively precise search strategy used in this study may limit the size of the corpus. We will expand our corpus from other platforms to train a more comprehensive topic model. Also, as a formative study, more traditional measures of consumers’ behavior are needed in addition to study on other social media platforms. Therefore, future research should consider analyzing more data on other social media platforms to explore users’ attitudes toward CBD more comprehensively.

### Conclusions

We collected posts related to CBD products from Reddit and Xiaohongshu and conducted theme mining using the LDA model. We analyzed the characteristics of the themes on social media and performed sentiment polarity on posts’ comments to compare users’ attitudes toward CBD products from different countries. The results showed that consumers in countries with different substance management policies had significantly different attitudes toward CBD. Therefore, more targeted CBD management measures should be formulated to suit each country’s national conditions. In addition, we hope that this study can provide a reference for other countries where CBD has not been approved for use in medicines, cosmetics, and food.

## Appendix

Multimedia Appendix 1 The visual layout of Latent Dirichlet Allocation (LDA) and perplexity and coherence with 5-fold cross-validation on documents of social media platforms.

## Abbreviations

CBD: cannabidiol
LDA: Latent Dirichlet Allocation
NLP: natural language processing
THC: tetrahydrocannabinol
