# Supplementary material for: Comparison of the Users’ Attitudes Toward Cannabidiol on Social Media Platforms: Topic Modeling Study
Source: JMIR Public Health Surveill. 2023 Jan 11;9:e34132. doi: 10.2196/34132 (PMC9878368; doi:10.2196/34132)
Supplement: Multimedia Appendix 1 [file publichealth_v9i1e34132_app1.docx]

## Multimedia Appendix 1

##
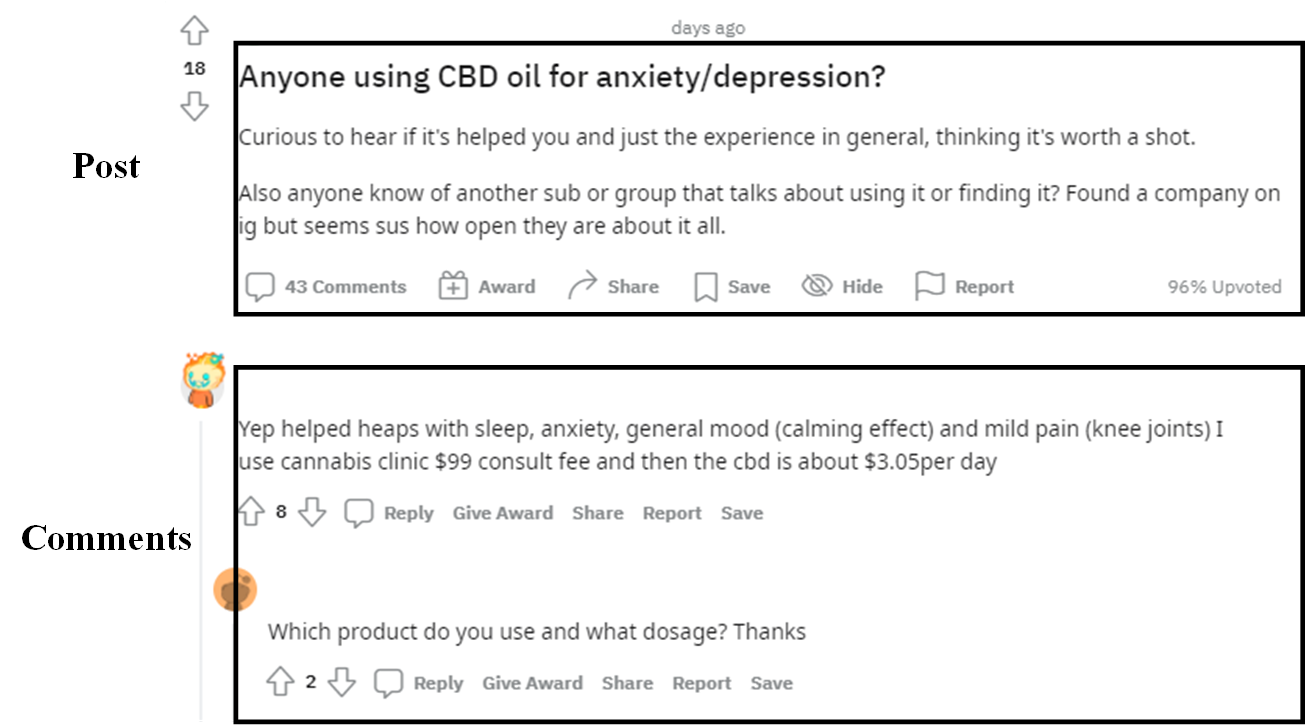


Figure S1. The difference between a post and comments.


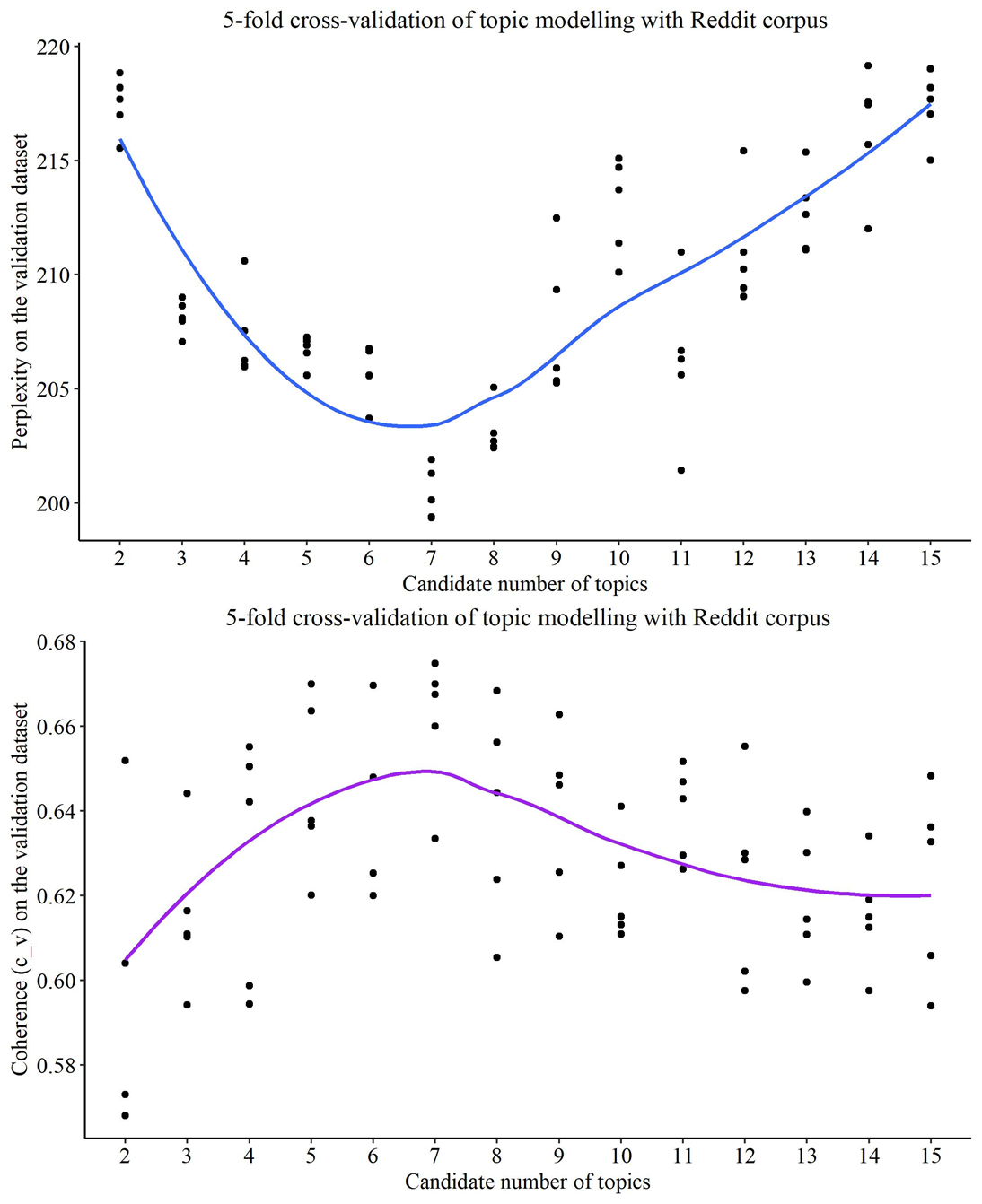


Figure S2. The results of perplexity and coherence with 5-fold cross-validation on Reddit documents.


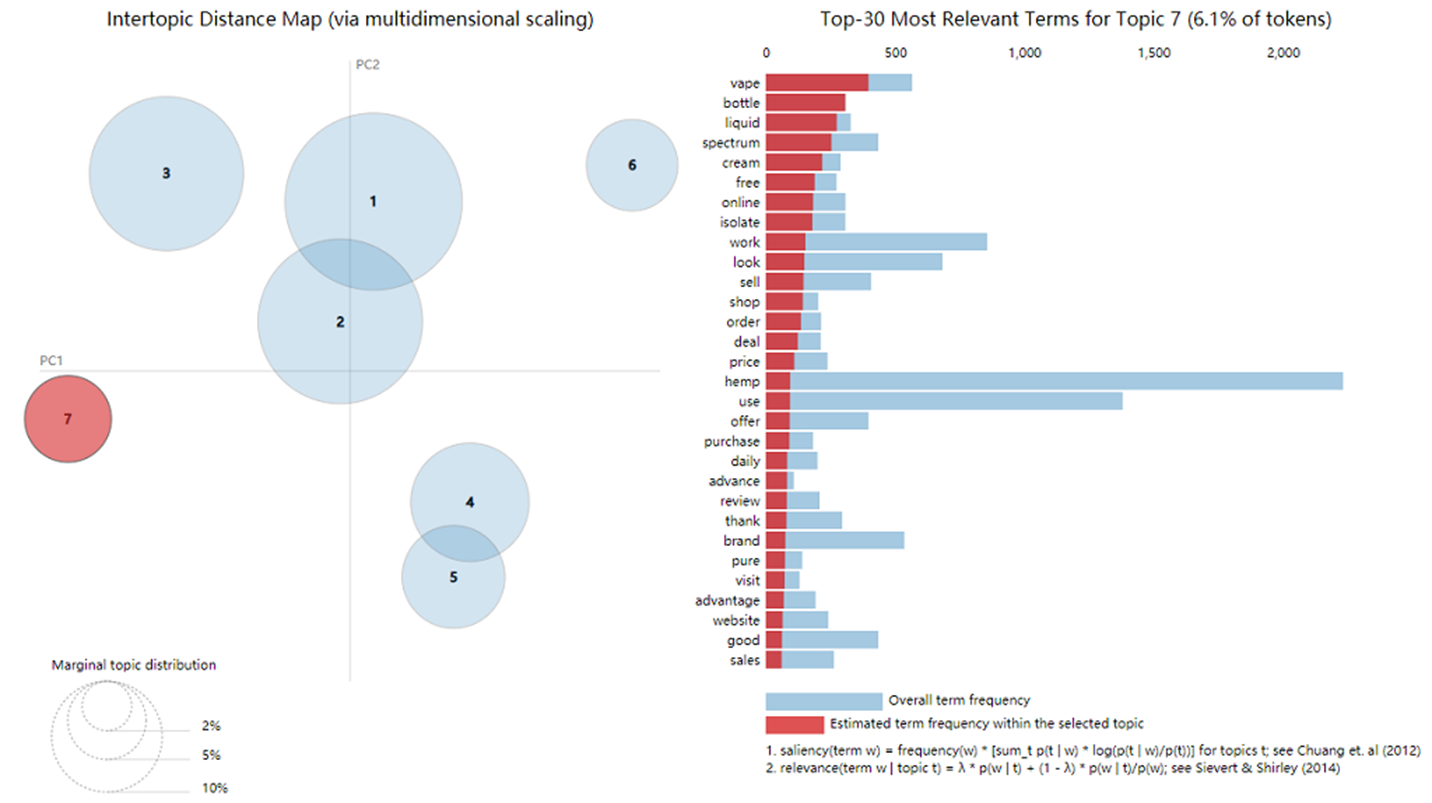


Figure S3. The layout of latent Dirichlet allocation on Reddit documents.


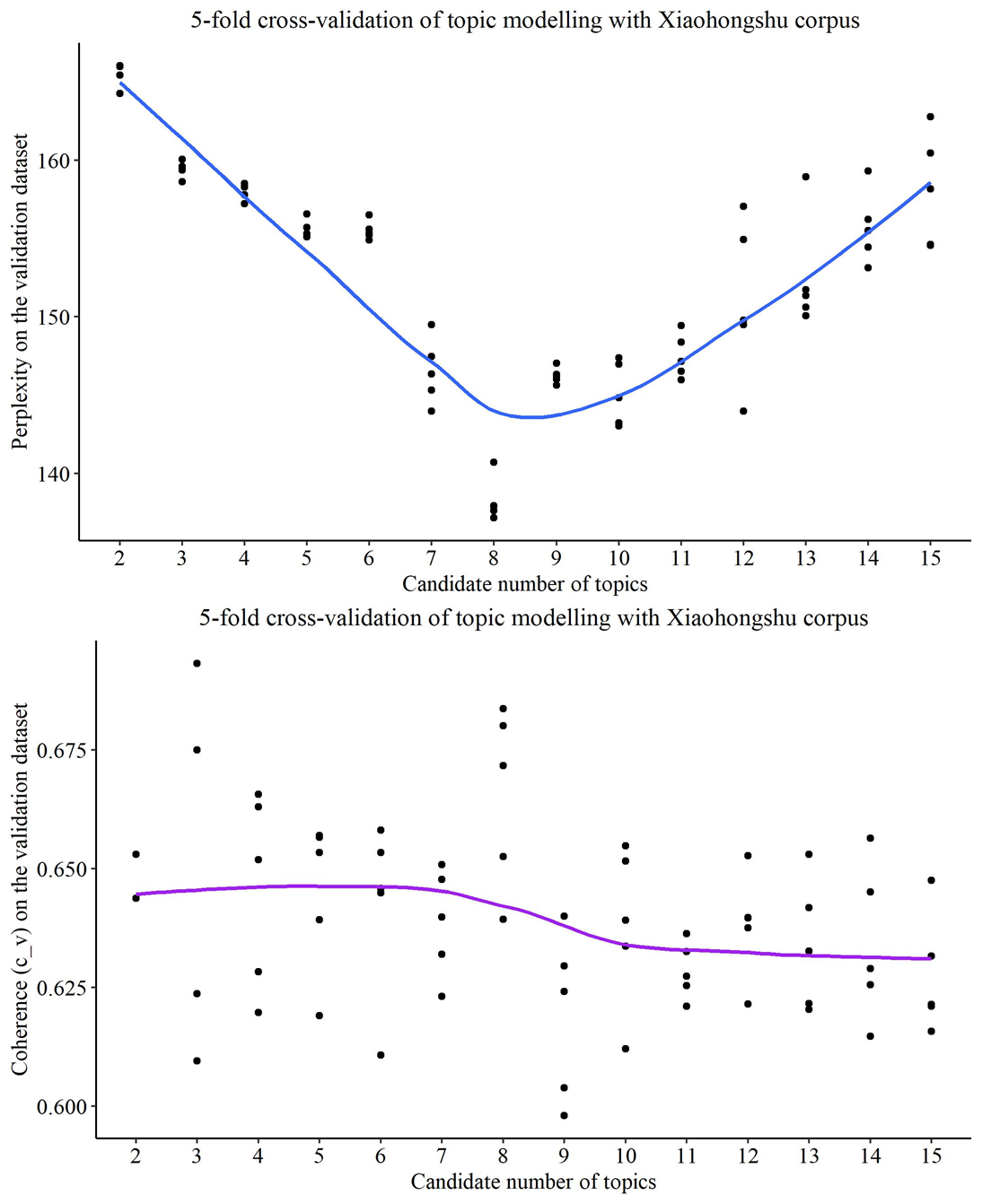


Figure S4. The results of perplexity and coherence with 5-fold cross-validation on Xiaohongshu documents.


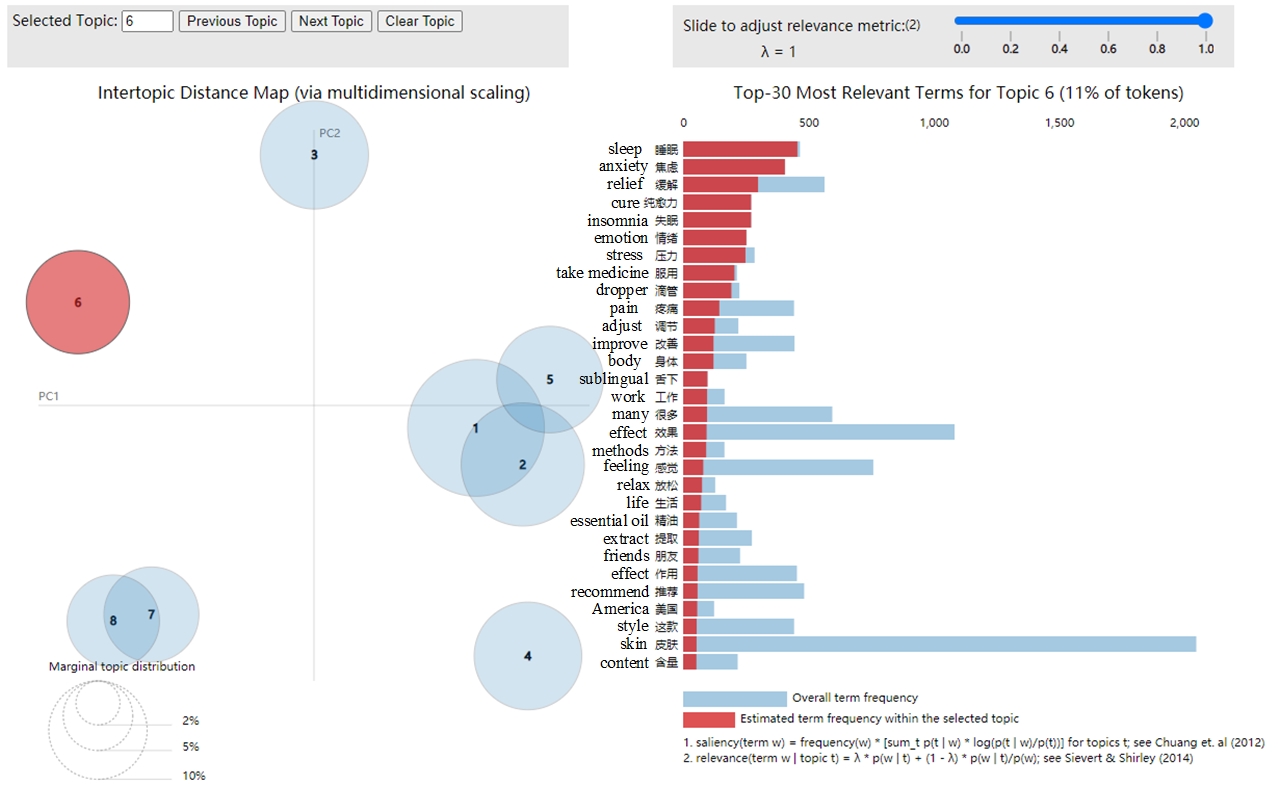


Figure S5. The layout of latent Dirichlet allocation on Xiaohongshu documents.
